# *In Situ* Atom Scale Visualization of Domain Wall Dynamics in VO_2_ Insulator-Metal Phase Transition

**DOI:** 10.1038/srep06544

**Published:** 2014-10-08

**Authors:** Xinfeng He, Tao Xu, Xiaofeng Xu, Yijie Zeng, Jing Xu, Litao Sun, Chunrui Wang, Huaizhong Xing, Binhe Wu, Aijiang Lu, Dingquan Liu, Xiaoshuang Chen, Junhao Chu

**Affiliations:** 1 Department of Applied Physics, Donghua University, No. 2999, North Renmin Road, Songjiang District, Shanghai 201620, China; 2 SEU-FEI Nano-Pico Center, Key Laboratory of MEMS of Ministry of Education, School of Electronic Science and Engineering, Southeast University, Nanjing 210096, China; 3 Optical Coatings and Materials Department, Chinese Academy of Sciences, Shanghai Institute of Technical Physics, No. 500, Yutian Road, Shanghai 200083, China; 4 National Laboratory for Infrared Physics, Chinese Academy of Sciences, Shanghai Institute of Technical Physics, No. 500 Yutian Road, Shanghai 200083, China; 5 These authors contributed equally to this work.

## Abstract

A domain wall, as a device, can bring about a revolution in developing manipulation of semiconductor heterostructures devices at the atom scale. However, it is a challenge for these new devices to control domain wall motion through insulator-metal transition of correlated-electron materials. To fully understand and harness this motion, it requires visualization of domain wall dynamics in real space. Here, domain wall dynamics in VO_2_ insulator-metal phase transition was observed directly by *in situ* TEM at atom scale. Experimental results depict atom scale evolution of domain morphologies and domain wall exact positions in (202) and (040) planes referring to rutile structure at 50°C. In addition, microscopic mechanism of domain wall dynamics and accurate lattice basis vector relationship of two domains were investigated with the assistance of X-ray diffraction, *ab initio* calculations and image simulations. This work offers a route to atom scale tunable heterostructure device application.

In semiconductor heterostructures, the interface is the device^1^,^2^ As development in semiconductor technology is propelling dimensions of devices down to atom scale, this description is becoming increasingly truthful^3^,^4^,^5^. Today semiconductor heterostructures rely on interfaces not only between different materials but also between different domains in the same material^6^,^7^. A domain wall is used as a device which can demonstrate many amazing varieties of electronic and optical properties, given device sizes can be smaller and domain wall location be controlled^8^,^9^. It is possible to bring about a revolution for tunable optoelectronic and microelectronic atom scale device application, based on efficient manipulation of domain walls through insulator-metal transition with correlated-electron materials^10^,^11^,^12^,^13^,^14^. However, the development of these new devices has been probably hampered by the lack of understanding of atom scale domain walls motion during insulator-metal transition process^15^,^16^,^17^,^18^.

In this paper, we laid emphasis on a typical correlated-electron material VO_2_^19^, with key feature of a first-order insulator–metal phase transition from the low-temperature monoclinic (M) phase to the high-temperature rutile (R) phase (Fig. 1a) at around room temperature. Despite it has characteristics of early discovery, convenient transition temperature and comparatively simple structures, the dynamic phase transition process still is not observed directly at atom scale^19^,^20^,^21^,^22^,^23^. Previous works only focused on characteristics of initial and final static states in phase transition process^10^,^11^,^14^,^19^,^20^,^21^,^22^,^23^. Here, we use an aberration-corrected transmission electron microscopy, which is extended to the limit of atomic scale, to directly observe domain wall dynamics. The experiments with high resolution images make unprecedented forms of information regarding atomic scale structural features accessible during dynamic phase transition process. In addition, temperature–dependent X-ray diffraction (XRD) is used to characterize and analyze domain wall dynamics. Empirical examinations with high resolution images and more quantitative analyses of integrated peak position profiles, when developed in conjunction with theory-supported modeling, provide deep insights into atomic level features of domain wall dynamics in VO_2_ phase transition.

*In situ* HRTEM images were obtained on FEI Titan 80–300, which operates with the nanocrystalline VO_2_ fabricated by a thermal oxidation method (see Supplementary Information). VO_2_ were mounted on a designed heating stage and were heated to the desired temperature (Methods). VO_2_ were mounted on a designed heating stage and were heated to the desired temperature (Methods). Fig. 1b shows a series of VO_2_ HRTEM images during the heating process. At room temperature (25°C), we observed, as expected, a HRTEM image with a corresponding fast Fourier transform (FFT) for <001> zone axis in monoclinic VO_2_ phase. As temperature increase to 50°C, the domain of monoclinic VO_2_ phase gradually decreases and that of rutile VO_2_ phase starts to emerge. The coexistence of monoclinic and rutile VO_2_ phases is clearly seen, which exhibits a first-order insulator-metal phase transition. Corresponding selected area FFT images can be used to identify domain phase categories. Thus, the changes of corresponding FFT images observed in Fig. 1b (image1, 2, 3, 4 and 5 labeled by the white lines) clearly presents domain wall motion process. Fig. 1c illustrates the schematic diagrams of VO_2_ band structure of monoclinic (insulator) and rutile (metal) phases before they form domain walls^18^,^24^,^25^. Fig. 1d shows energy band change diagram for tunable heterostructures, accompanied by domain wall motion. To understand detailed structural characterization of domain wall dynamic process, we analysis three representative *in situ* HRTEM images (image 1, 2 and 5 in Fig. 1b) before VO_2_ phase transition (Fig. 2a), the HRTEM image of two-phase coexistence during VO_2_ phase transition((Fig. 2b), and that after VO_2_ phase transition(Fig. 2c). The simulated electron diffraction patterns (Methods) show that these patterns are for <001> zone axis of monoclinic structure (indicated by ii in Fig. 2a) and <
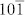
> zone axis of rutile structure (indicated by ii in Fig. 2c), which match well with that of FFT images obtained at temperature of 25°C (indicated by i in Fig. 2a) and 70°C (indicated by i in Fig. 2c), separately. In Fig. 2b, the selected area FFT images show two sets of diffraction spot, which are assigned to monoclinic and rutile phase, respectively. The domain walls are marked by a white dotted line in Fig. 2b. Symmetrical lattice fringes with interplanar distance of 4.86 and 4.60 Å can be observed in Fig. 2a, which can be indexed as (100) plane and (010) plane of monoclinic VO_2_. In Fig. 2c, the interplanar distance of 4.61 and 2.14 Å can be indexed as (010) plane and (111) plane of rutile VO_2_, respectively. Geometrical phase analysis (GPA) of Fig. 2b is also used to calculate the strain map around the domain wall^26^. GPA obtains the corresponding strain field relative to some presumably unstrained area of the HREM image. The results of the strain components ε_xx_ and ε_yy_ are shown in Fig. 2d, respectively. The variation of rutile phase domain indicates the strain field change, which can be noticed that there are several convergence regions of strain. Conversely, the monoclinic phase presents a homogeneous strain distribution.

In order to categorically identify phase interface and atom structure changes, atom structure models were applied in HRTEM analysis. Fig. 3a shows a magnified view of Fig. 2b, which is smoothed for reducing noise (not affecting interpretation of atomic position)^27^. To distinguish phase structure changes, we used simulated HRTEM images to compare predicted contrast variations. By adjusting image defocus and resolution (see Methods), HRTEM image simulations can characterize domain walls in VO_2_ phase transition. In Fig. 2b, region I and II represent simulated rutile and monoclinic phase HRTEM images, separately^28^. These simulated HRTEM images match well with our experimental HRTEM images. The simulated HRTEM images indicate that rutile phase owns bright continuous dots, which are marked in Fig. 2b region I. However, monoclinic phase has bright interrupted dots (in region II of Fig. 2b). This directly shows differences between VO_2_ rutile and monoclinic phases. By enlarging coexisted rutile and monoclinic phase region, the positions of domain walls (white solid line) are clearly localized in Fig. 3a. The rutile phase structure is on the left, whereas monoclinic phase structure is on the right. The domain walls are in (202) and (040) planes of rutile structure, which is consistent with earlier results^29^,^30^. Taking into account that the upper surface is a (001) plane of the monoclinic phase, the angles between domain walls and the direction of monoclinic VO_2_ phase b_M_ axis are 0°and 90°. The <010> axis of monoclinic VO_2_ phase corresponds to a <
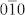
> direction of rutile VO_2_ phase. The color inserts in Fig. 3a correspond to atomic structure model images. The red and black spheres represent O and V atoms, respectively. In inserts, the atomic arrangements of two phases have obvious differences, which are shown in green dotted and solid rectangles, respectively. In Fig. 3a, a V atom and another V atom, which we call V-V pair, are found to have relative motion, but there is no relative motion between O atoms during VO_2_ phase transition. In Fig. 3a, experimental lattice parameters of inter-relationship between rutile and monoclinic domains were obtained from corresponding domain FFT images (Fig. S3). The interplanar distance indexed as (100) plane of monoclinic domain was 4.862 Å, the same as corresponding distance in rutile domain. Monoclinic (010) plane with inter-plane spacing of 4.604 Å was equivalent to corresponding distance of rutile phase (010) plane. These results indicate that there was no expansion perpendicular to c_M_ axis, where M refers to monoclinic phase.

*In situ* HRTEM images show no expansion perpendicular to c_M_ axis, to confirm the expansion along c_M_ axis, temperature-dependent XRD measurements were performed using a Siemens-Brucker D8DISCOVER diffractometer with the X-ray cathode source of *CuKα* (λ = 1.5406 Å). Fig. 4a shows monoclinic VO_2_ (002)_M_ peaks shifting to (200)_R_ peaks of rutile phase during heating process, which is consistent with atomic structure models in Fig. 4b. The lattice parameters in heating process can be estimated by means of Bragg's law, which is expressed as 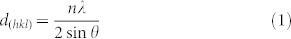
where *λ* is wavelength of X-ray, *θ* is scattering angle, *n* is an integer representing order of XRD peak. The lattice parameters (Fig. 4c) can be calculated on the basis of structural characteristic and similarities between monoclinic and rutile phases shown in Fig. 4b. The calculated values are obtained from data in Fig. 4a (marked as i and ii ) and β is 122.6° (Table S1 in Supplementary Information). The results show a tiny expansion between two domains.

The crystal fine-structure in VO_2_ phase transition is discussed further. Dynamic experiments show direct accurate relationship of lattice basis vectors at initial (monoclinic phase), coexisting (monoclinic/rutile phase) and final (rutile phase) states. These are the basis of theoretical and experimental explorations in VO_2_ phase transition^31^,^32^,^33^. For example, Cs-corrected scanning transmission electron microscopy (STEM) is recently performed to investigate microstructures of the epitaxial polycrystalline VO_2_ thin films^34^. The atomic resolved STEM experiments are done at room temperature. However, corresponding high temperature rutile structure experiment has not been done and its structure is only deduced from the relationship of two phase lattice basis vectors. These works only researched static initial (monoclinic phase) and final (rutile phase) lattice basis vector relationship^31^. At static experiments, there are many matching results of monoclinic and rutile crystal orientations, which is very difficult to find accurate matching relationships. But *in situ* HRTEM atom scale dynamic experiments makes it possible for accurately and directly identifying crystal structure relationships of two phases. Corresponding with initial, coexisting and final HRTEM images in Fig. 2a to 2c, a schematic illustration of this crystal structure variation at atomic level is shown in Fig. 3b and 4c. To explain detailed structure changes, a unit VO_2_ structure diagram is shown in Fig. 5a. The figure clearly shows V-V pair positions of two stable structures. A V atom and another V atom can be bound together to form V-V pair through chemical bonding with two O atoms. During phase transition, motion of V atoms is from the initial gray V_1_ and V_2_ positions to the final green V_1_′ and V_2_′ positions, respectively. V-V pair undergoes not only elongation from 2*d_1_* (distance of two gray V atoms) to 2*d_2_* (distance of two green V atoms) but also a twist *θ* angle in X-Z plane. Fig. 5b shows a three dimensional schematic view of inter-relationship between rutile and monoclinic structures. In Fig. 5c, three-view depictions show accurate lattice basis vectors relationships of monoclinic and rutile phases. Viewed from rutile <010> zone axis direction, monoclinic (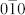)_M_ plane is turned into rutile (010)_R_ plane (marker i to i′ in Fig. 5c). Along rutile <
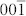
> and <100> zone axis direction, monoclinic (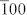)_M_ and (001)_M_ planes are turned into rutile (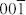)_R_ and (100)_R_ planes (from ii, iii to ii′, iii′ in Fig. 5c), respectively. Contrary to previous reports^30^,^34^, the corresponding structural relationship of rutile and monoclinic phases can be written as 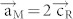, 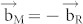, 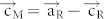 by our dynamic HRTEM experiments. This provides a direct evidence of these two phases spatial relationship during the insulator-metal phase transition, which is important in clarifying the mechanism of VO_2_ phase transition.

To understand domain wall dynamical process, real-time observation of experimental phase transitions in structured variants with high spatial resolution is needed to be conducted. In fact, atom movement spatial scale, temporal resolution and domain wall positions are crucial to understand and harness VO_2_ domain wall dynamics. In previous work, Peter Baum and colleagues have shown the temporal displacements of atoms in picoseconds, that the V-V bond dilation is the initial step of the insulator-metal transition and an long-range shear rearrangements follows the V-V movement^35^,^36^. In this study, we use the high-resolution TEM to directly elucidate the spatial VO_2_ domain wall dynamical process at the atomic level. The atom scale exact domain wall positions have been also observed. On the other hand, we present the clear direct experimental evidence that V atomic motion in VO_2_ phase transition from the initial to the final position forms the V-V pair movement, which is concordantly predicted by numerous theoretical treatments. In addition, microscopic mechanism of domain wall dynamics in VO_2_ phase transition is also investigated. We propose a possible two-step process (Fig. 6a) of domain wall motion: first step is movement of V-V pairs and second step is expansion along c_M_ axis. First, at room temperature primary rutile phase VO_2_ nucleates on defect domain of monoclinic phase, as defect domain possess enough free electrons^37^,^38^. Then a Schottky junction is formed at monoclinic/rutile domain walls shown in Fig. 6b. Previous reports have shown band bending on the domain wall can alter the spatial distribution of electron concentration and VO_2_ phase transition behavior^24^,^39^. Electric field at the domain walls produces a force on electrons, which prevents electrons diffusion from rutile to monoclinic phase domains. Simultaneously, the further extend of rutile phase domain is suspended. When the sample is heated, thermal equilibrium of domain walls loses at two sides, some electrons of rutile phase domain are injected into monoclinic phase domain^20^,^22^,^40^. This may strengthen electron-phonon interactions and electron-electron interactions^41^, which drives the movement of V-V pairs (Fig. 5a). When the sample reaches its phase transition temperature, many electrons of rutile phase domain can cross over Schottky battier of domain walls to monoclinic phase domain. This process can be described by the Richardson equation, written as^42^

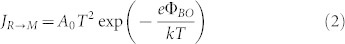
where *J_R→M_* is thermionic emission electron current density, *A_0_* is Richardson constant which is equal to 120 A/(cm^2^·K^2^) for electrons, *T* is temperature, *eΦ_BO_* is Schottky barrier height, and *k* is Boltzmann constant. The thermionic emission electron current densities depending on temperature are calculated when Schottky barriers height is 0.11 eV shown Fig. 6c, and results show thermionic emission electrons current densities rapidly increasing at approximately 62°C. The Schottky barrier height of this model is consistent with earlier measurements^24^. A schematic illustration is shown in Fig. 6d.

Second, when movement of V-V pairs is completed, the whole structure expands along c_M_ axis to form final stable rutile phase state. The XRD results show that a tiny expansion of approximately 0.023 Å (Fig. 6e) occurs during phase transition. When movement of V-V pairs is finished, this structure will possess a higher lattice potential than final stable VO_2_ rutile sructure^43^,^44^. As separation of two adjacent V-V pairs along c_M_ axis can decrease lattice potential, the expansion along this direction occurs and form final stable VO_2_ rutile phase structure, as shown in Fig. 6a.

In summary, we directly observed domain wall dynamics in VO_2_ phase transition using *in situ* HRTEM at the atom scale. In contrast to no expansion perpendicular to c_M_ axis between two domains, a tiny expansion of approximately 0.023 Å is found along c_M_ axis. Domain wall positions are exactly located in (202) and (040) planes of rutile structure at the temperature of 50°C. Microscopic mechanism of domain wall dynamics is also analyzed. The structure analysis offers fine-structure views at initial (monoclinic phase), coexisting (monoclinic/rutile phase) and final (rutile phase) states. The corresponding structural relationship of rutile and monoclinic phases is written as 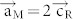, 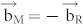, 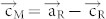. More efforts will still be required to clarify comprehensive theoretical description of VO_2_ insulator–metal phase transition. Nonetheless, the fine-structure information and accurate relationship of lattice basis vectors presented here can supply a structural framework for theoretical and experimental further explorations in VO_2_ phase transition^45^,^46^. The work can be used to design and engineer atom scale heterostructures devices. Crucially, this treatment method of domain wall used as a device can make us to dynamically modify domain walls even after the assembly into device architecture, and also plays an important role in overcoming device size limit when individual element dimensions in devices continue to shrink^47^,^48^.

## Methods

The *in situ* HRTEM images were obtained with an image aberration-corrected microscope (FEI Titan 80–300 operating at 300 kV). A charge-coupled device camera (2 k × 2 k, Gatan UltraScan 1000) was used for image recording with an exposure time of 1 s to 2 s. The third-order spherical aberration was set in the range of 10 μm to 20 μm, and the TEM images were recorded under slightly defocused condition. The heating was conducted using a heating sample holder (Gatan 628). To ensure that the sample temperature was consistent with that of the measured temperature, we waited for at least 30 min to achieve thermal equilibrium before further imaging. The electron diffraction patterns were simulated by means of CRYSTALMAKER software packages with the value from the theoretical simulation (Supplementary Information). The atomic models of the monoclinic phase and rutile phase were created via Accelrys Discovery Studio Visualizer^27^ and the corresponding simulation of the HRTEM images were performed by means of the multislice algorithm with parameters set in accordance to the approximations for the microscope^28^.

## Author Contributions

X.F.H. prepared the VO_2_ sample. T.X. performed TEM imaging and analyzed the data. Y.J.Z. and H.Z.X. carried out the theoretical investigation. X.F.H. and J.X. conducted the structure analyses. C.R.W., D.Q.L., A.J.L. and B.H.W. contributed to the discussion and interpretation of the results. J.H.C. supervised the project and provided guidance. X.F.H., X.F.X., L.T.S. and X.S.C. organized and wrote the manuscript with input from all authors.

## Supplementary Material

Supplementary Information

## Figures and Tables

**Figure 1:**
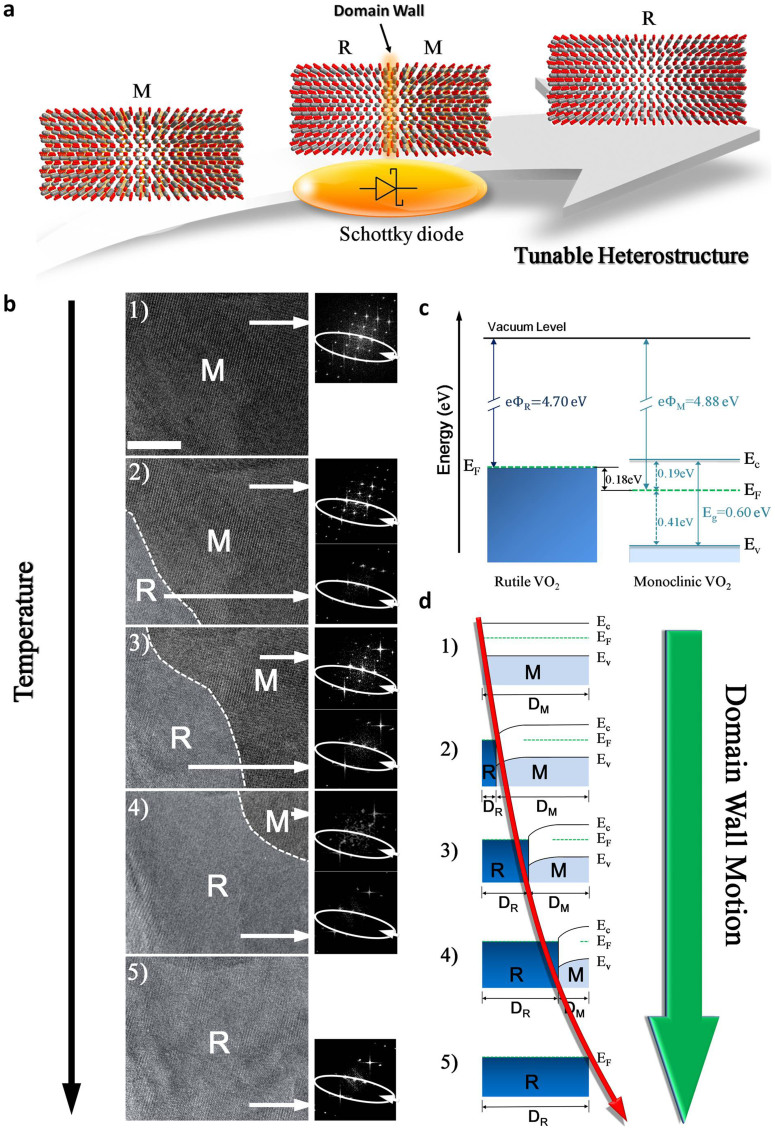
Domain wall dynamics. (a), Schematic illustration of domain wall dynamics in VO_2_ insulator-metal phase transition (from monoclinic (M) phase to rutile (R) phase) for the atom scale tunable heterostructure. The red and gray spheres represent O and V atoms, respectively. (b), A series of *in situ* HRTEM images with corresponding fast Fourier transformation (FFT) images at temperature of 25, 50, 55, 60and70°C (from images 1 to 5) show domain wall dynamics in VO_2_ insulator-metal phase transition. (c), Energy band diagrams of rutile and monoclinic VO_2_ before formation of domain walls. (d), Schematic energy band change diagram for tunable heterostructure, where D_M_ and D_R_ are widths of monoclinic (M) and rutile (R) domains. In (b) scale bars are 10 nm.

**Figure 2:**
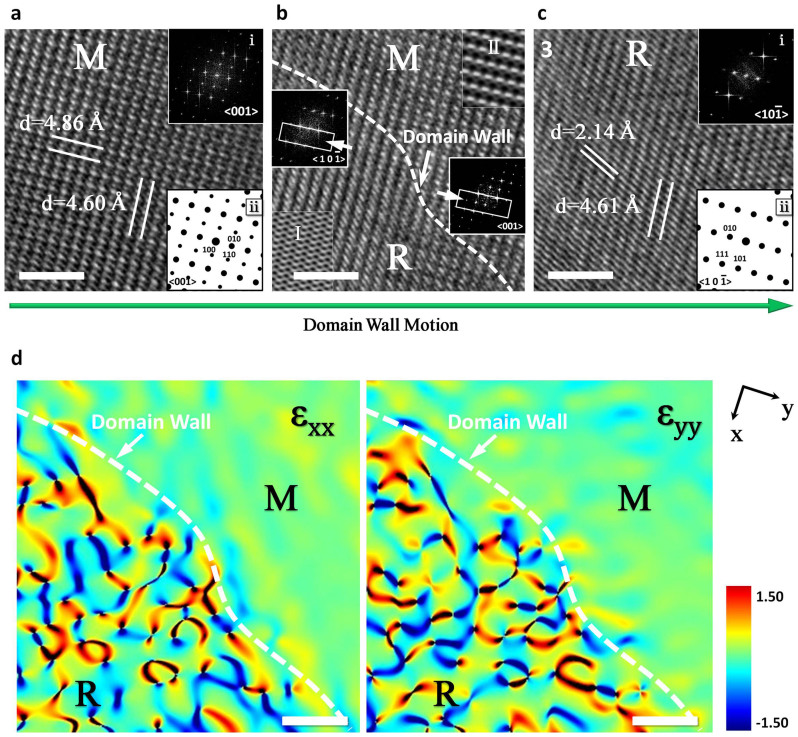
Sequences of three representative in situ HRTEM images. (a), *In situ* HRTEM image of monoclinic VO_2_ at 25°C. The corresponding FFT images (i) and simulated electron diffraction patterns (ii) are shown in inset. (b), *In situ* HRTEM image at 50°C. Domain wall is indicated by a white dotted line. Insets show comparison between experimental HRTEM micrographs and simulated TEM micrographs (labelled by the white rectangle) of rutile (I) and monoclinic (II) phase. (c), *In situ* HRTEM image of rutile VO_2_ at 70°C. The corresponding FFT image (i) and simulated electron diffraction patterns (ii) are shown in inset. (d), Experimental strain components ε_xx_ and ε_yy_ obtained by geometric phase analysis (GPA) of (b). In (a–d) scale bars are 2 nm.

**Figure 3:**
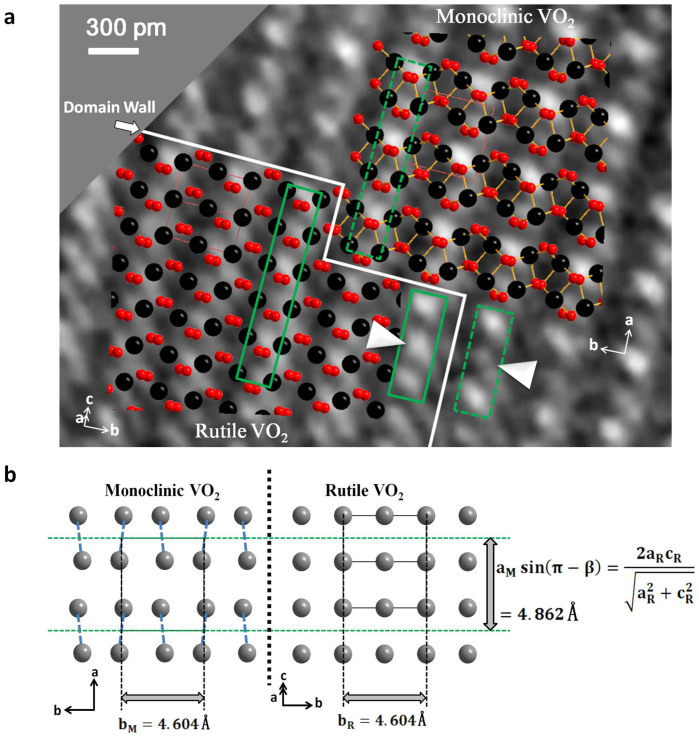
An enlarged view of domain wall. (a), A smoothed HRTEM image of monoclinic/rutile domain walls. Insets show two-phase color atom model diagrams. The red and black spheres represent O and V atoms, respectively. (b), The lattice parameters of the inter-relationship between rutile and monoclinic domains perpendicular to c_M_ axis; for clarity, only V atoms are shown. The dashed green lines are guided to eye with respect to unit cells.

**Figure 4:**
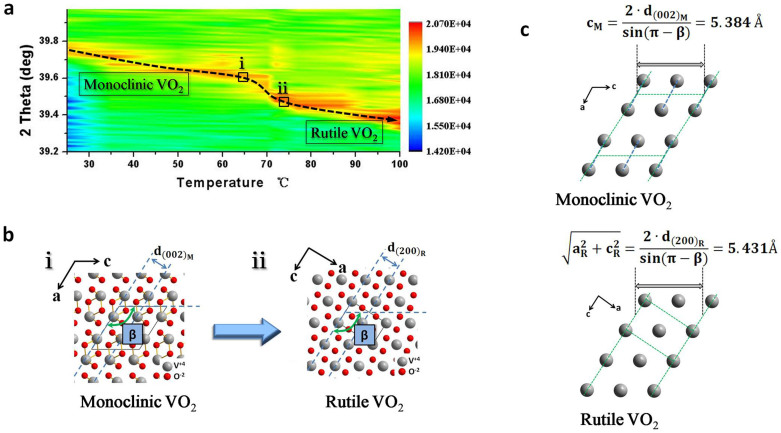
Expansion along to c_M_ axis. (a), The temperature-dependent XRD spectra. (b), Corresponding atomic structure models show changes in XRD pattern with an increase in temperature. (c), Along c_M_ axis, lattice parameter relationship between rutile and monoclinic domains; for clarity, only V atoms are shown.

**Figure 5:**
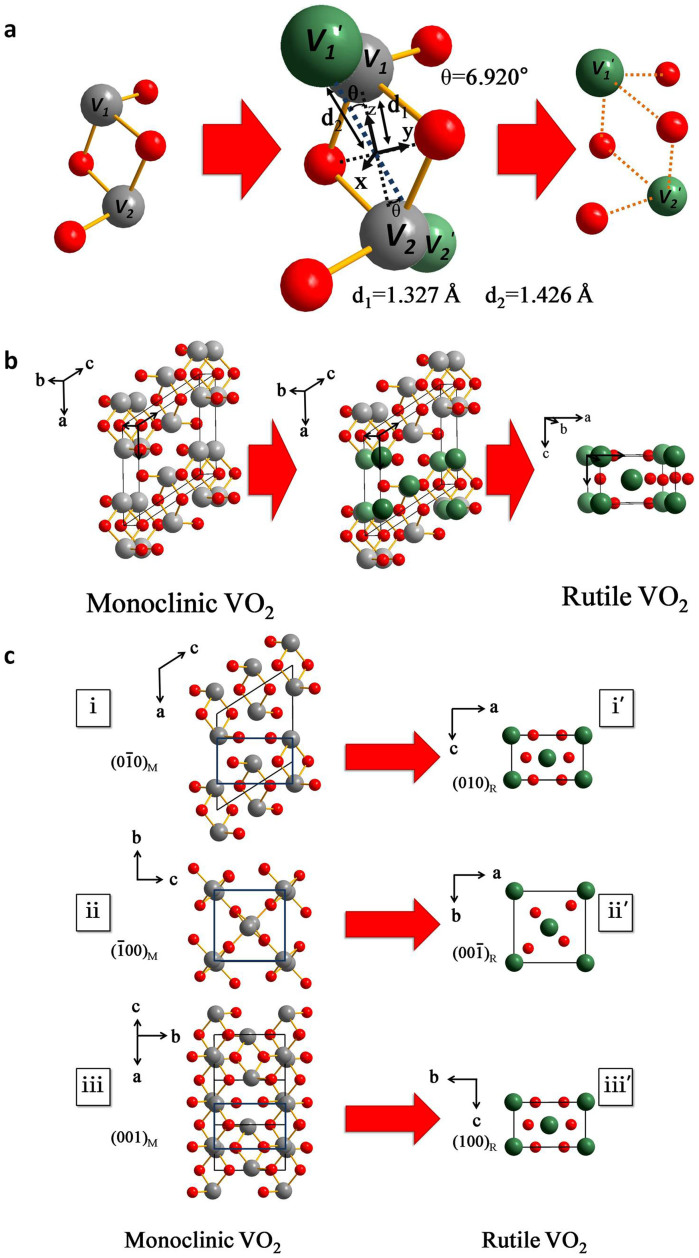
A schematic of inter-relationship between rutile and monoclinic phases. (a), Schematic of a V-V pair movement. Movements of V atoms are from initial (gray) to final (green) positions. (b), A three dimensional schematic of inter-relationship between rutile and monoclinic phases. (c), Three-view depictions of phase transition in VO_2_ from monoclinic phase (left) to rutile phase (right). Red and gray (green) spheres respectively represent O and V atoms.

**Figure 6:**
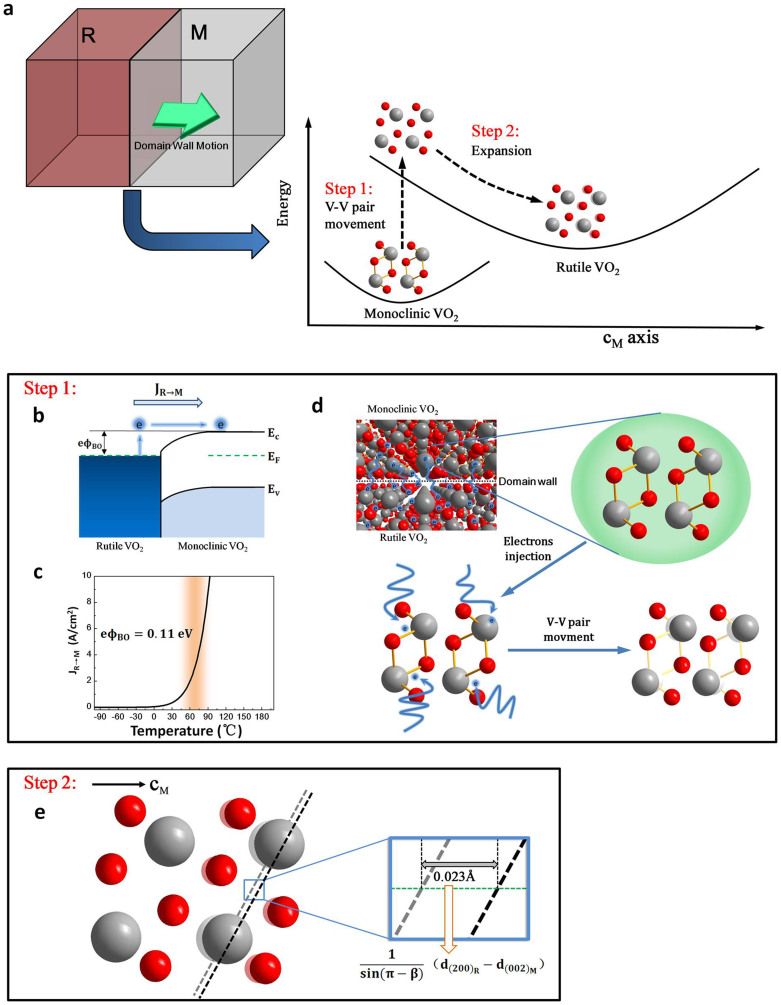
Microscopic mechanism of domain wall dynamics. (a), Schematic energy diagram of domain wall motion. (b), The energy-band diagram of a Schottky junction at monoclinic/rutile domain walls. (c), The thermionic emission electron current density *J_R→M_* as a function of temperature with a Schottky barrier of 0.11 eV. (d), An illustration of V-V pair movement in first step. (e), Expansion diagram along c_M_ axis in second step. The red and gray spheres represent O and V atoms, respectively.
