# Supplementary material for: In Situ Atom Scale Visualization of Domain Wall Dynamics in VO2 Insulator-Metal Phase Transition
Source: Sci Rep. 2014 Oct 8;4:6544. doi: 10.1038/srep06544 (PMC4189024; doi:10.1038/srep06544)
Supplement: Supplementary Information [file srep06544-s1.pdf]

Supplementary Information

for

In Situ Atom Scale Visualization of Domain Wall Dynamics in VO_2 Insulator-Metal Phase Transition

Xinfeng He[1], Tao Xu[2], Xiaofeng Xu[1★], Yijie Zeng[1], Jing Xu[1], Litao Sun[2★], Chunrui Wang[1], Huaizhong Xing[1], Binhe Wu[1], Aijiang Lu[1], Dingquan Liu[3], Xiaoshuang Chen[4★], Junhao Chu[4]

[1] Department of Applied Physics, Donghua University, No.2999, North Renmin Road, Songjiang District, Shanghai 201620, China.[2] SEU-FEI Nano-Pico Center, Key Laboratory of MEMS of Ministry of Education, School of Electronic Science and Engineering, Southeast University, Nanjing 210096, China. [3]Optical Coatings and Materials Department, Chinese Academy of Sciences, Shanghai Institute of Technical Physics, No.500, Yutian Road, Shanghai 200083, China. [4]National Laboratory for Infrared Physics, Chinese Academy of Sciences, Shanghai Institute of Technical Physics, No.500 Yutian Road, Shanghai 200083, China.

★E-mail: xxf@dhu.edu.cn; slt@seu.edu.cn; xschen@mail.sitp.ac.cn

1. Fabrication of the nanocrystalline VO_2

Similar to our previous work[1-3], a simple method of sputtering oxidation coupling (SOC) was used to fabricate VO_2. The metallic *V* was deposited on sapphire (c-plane) substrate by DC-sputtering deposition technique at room temperature. The vacuum chamber was evacuated down to 5.0×10^{-3} Pa and argon gas (99.99% purity) was set at 1.0 Pa. The samples were then oxidized at high temperature in the air atmosphere. By optimizing the oxidation time, VO_2 nanocrystals were successfully fabricated.

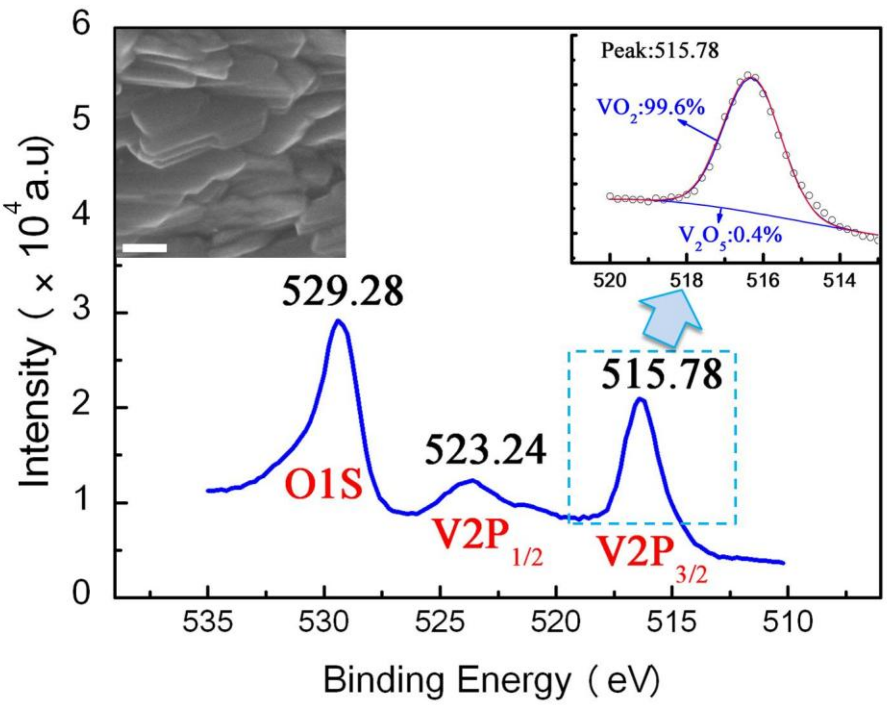

Figure S1 | Characterization of VO_2 using X-ray photoelectron spectroscopy (XPS). Up right inset is the enlarged image of the sample to show the detail in the blue dotted box: core-level XPS (open circles) and corresponding fitting result (solid lines). The upper left insert shows the SEM image of the sample; scale bar is 50 nm.

XPS experiments were performed by means of an RBD upgraded PHI-5000C ESCA system (PerkinElmer). The Mg Kα radiation (hν= 1253.6 eV) was operated at 14 kV and 20 mA. The carbonaceous $C1_S$ line (284.6 eV) was used as reference in calibrating the binding energies. Two typical peaks ($V2P_{1/2}$ and $V2P_{3/2}$) were clearly observed because of the orbital splitting (Fig.S1). The $V2P_{3/2}$ core-level peak appears at 515.78 eV, which is close to the well-established value for V^{+4}, indicating a single 4+ oxidation state of V^4. The peaks at 515.6 and 517.0 eV correspond to two valuences of vanadium V^{+4} and V^{+5} respectively. With XPS Peak Processing Software,

the molecular content of the elemental chemical state was obtained with the itergrated area of 515.6 and 517.0eV peaks [1,5]. The result shown in Fig. S1 (upperright inset) indicates that theVO_2 components of the sample are >99 %.

As a function of temperature, the electrical properties for the heating process were measured through four-point probe method; the samples were placed on a ceramic heater with temperature accuracy of ±0.1℃. The sheet resistance decreases gradually as temperature increases, which eventually reaches approximately four orders of magnitude drop (Fig.S2). The shaded area highlights the region of the two-phase mixture. The temperature-dependent resistance measurement indicates a classical insulator-metal transition in VO_2.

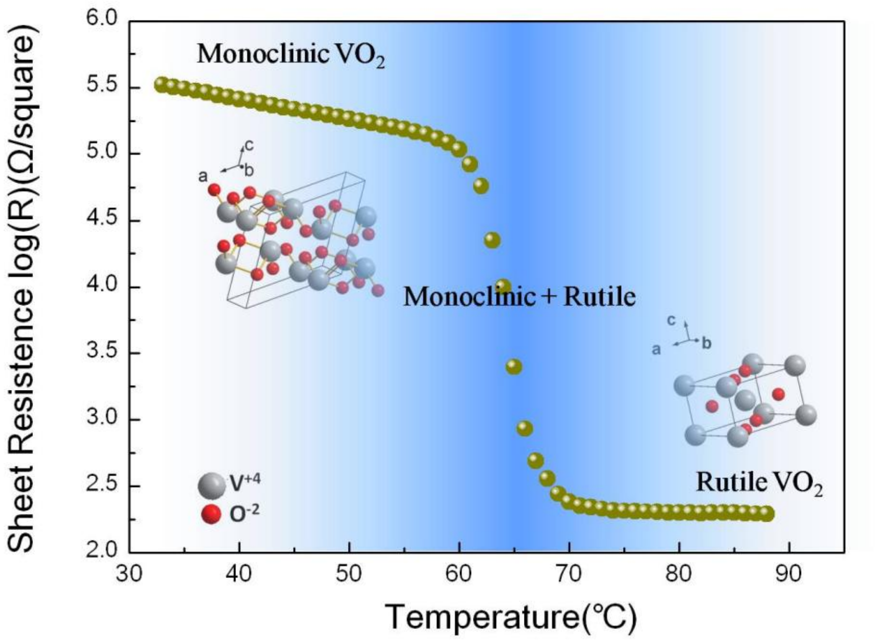

Figure S2 | Temperature-dependent sheet resistance changes of VO_2. The insets show the room-temperature and high-temperature crystal structures of the corresponding compounds.

To prepare the samples for HR-TEM experiments, nanocrystalline VO_2 was released from the sapphire surface, and then dispersed in anhydrous ethanol (0.1 wt %). The sample was then subjected to ultrasonication to achieve better dispersion. A drop of suspension was deposited onto a copper lacey support film, and then covered with a carbon film. The experiments were performed on a heating stage, which was heated or cooled at the desired temperatures.

2. The structural relationship of the monoclinic phase and rutile phase (perpendicular to c_M axis referring for monoclinic phase):

No expansion that is perpendicular to the monoclinic phase c_M axis was observed during the phase transition.

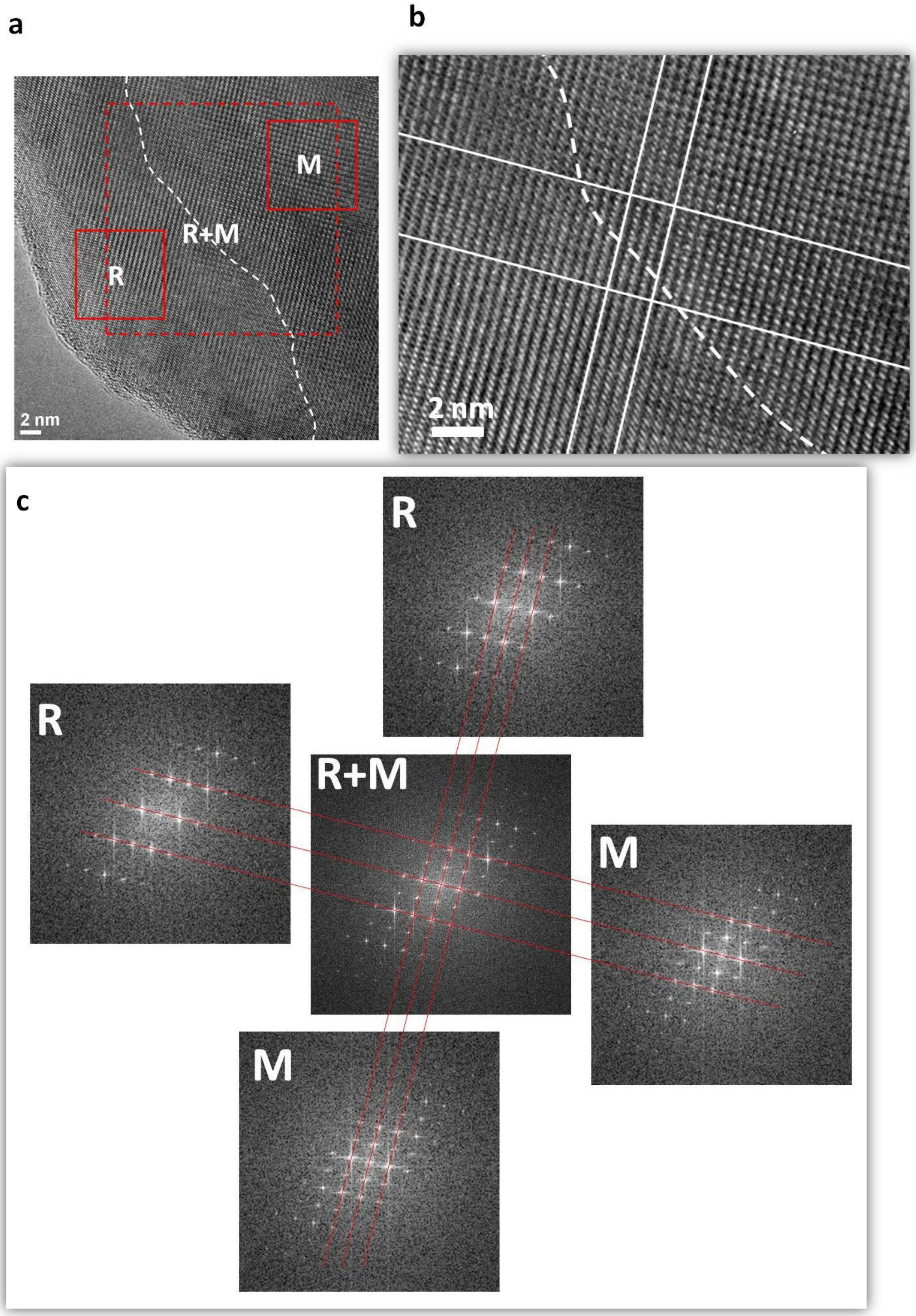

Figure S3｜The structure relationship of the monoclinic phase and rutile phase. a， HRTEM image of the sample at the temperature of 50℃. **b，** Enlarged view of the monoclinic phase /rutile phase interface in **a**. **c，** The corresponding FFT images of the domains marked as M,R and R+M in**(a)**.

3. *Ab initio* calculation:

We performed structural optimization of the monoclinic and rutile VO_2 based on the plane-wave pseudopotential density functional theory (DFT), which was implemented in the CASTEP code[6]. Generalized gradient approximation (GGA) with exchange-correlation functional was used following the approach of Perdew-Burke-Ernzer-hof (PBE)[7]. The electronic wave functions are described by a set of plane wave functions. The energy cut-off for the plane wave expansion was set to 360 eV, and a Monkhorst-Pack k-point mesh of $4\times4\times4$ is used for Brillouin zone integration. Convergence in energy and force was set to 2×10^{-5} eV/atom and 0.05eV/Å, respectively[6,8]. The optimized structures were used for electron density calculations of the monoclinic VO_2.

Fig.S4 and S5 show the calculated electron density map of the monoclinic phase and rutile phase, respectively. The red to blue colors represent positive (maximum) to negative (minimum) electron densities. The positions of V and O atoms are indicated as the red and gray spheres, respectively.

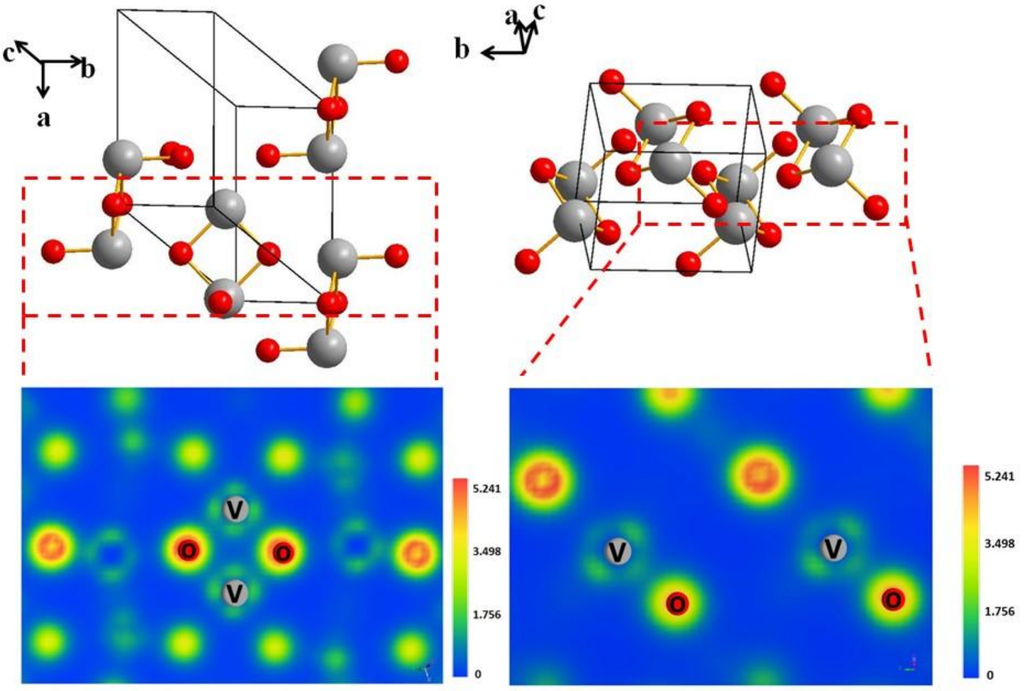

Figure S4｜ **Crystal structures and simulation electron density maps of the monoclinic VO_2; the red and gray spheres represent O and V atoms, respectively.**

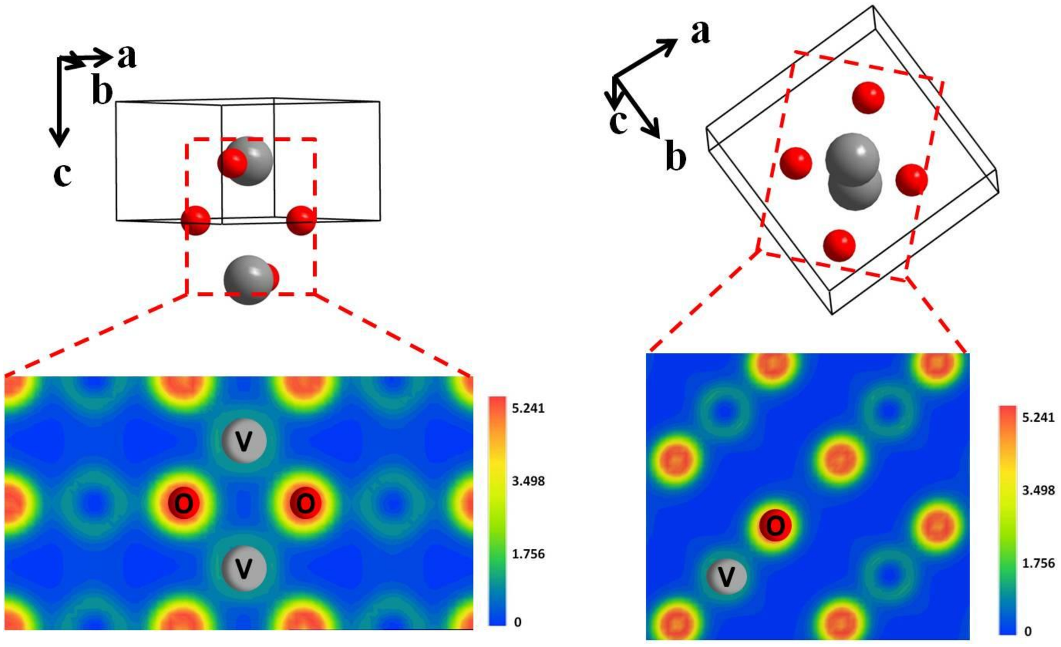

Figure S5｜ **Crystal structures and simulation electron density maps of the rutile VO_2; the red and gray spheres represent O and V atoms, respectively.**

Table S1 shows the experimental and theoretical lattice parameters, which are used

for comparison of the rutile phase and monoclinic phase.

Table S1 | Comparison between the lattice parameters in previous studies and those obtained in our study

			Present work	Renata M. Wentzcovit et al. [9]	Tao Yao et al. [10]	J.Cao et al . [11]
**Monoclinic VO_2**	**Lattice constants(Å)**	**a**	5.547	5.629	5.73109	5.75
		**b**	4.576	4.657	4.51112	4.54
		**c**	5.367	5.375	5.34158	5.38
	**Angle (°)**	**α**	121.59	121.56	122.635	122.6
		**β**	90	90	90	90
		**γ**	90	90	90	90
**Rutile VO_2**	**Lattice constants (Å)**	**a**	4.601	4.634	4.53085	4.55
		**b**	4.601	4.634	4.53085	4.55
		**c**	2.792	2.804	2.84883	2.85
	**Angle (°)**	**α**	90	90	90	90
		**β**	90	90	90	90
		**γ**	90	90	90	90

4. The thermionic emission current densities:

The thermionic emission electron current density curves as a function of the temperature *T* are calculated with different Schottky barriers, which are shown in Fig.S6. The saltation temperature of the thermionic emission electron is very sensitive to a slight variation in the Schottky barrier. A tiny change in the barrier can lead to a significant alternation of the saltation temperature, which may determine the phase transition temperature.

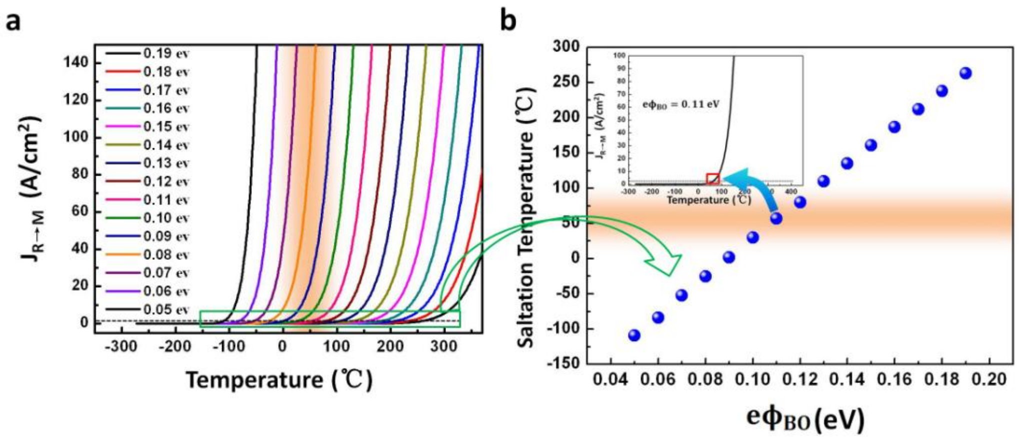

Figure S6|The thermionic emission current. a，The thermionic emission electron current density $J_{R\to M}$ as a function of the temperature with different Schottky barriers from 0.05 eV to 0.19 eV. **b，** The Schottky barrier dependence of the saltation temperature. The inset image shows thermionic emission electron current density for the Schottky barrier of 0.11 eV.

Supporting Information References

1. Xiaofeng Xu et al. A novel sputtering oxidation coupling (SOC) method to fabricate VO_2 thin film. Applied Surface Science **256** 2750–2753(2010)

2. Xiaofeng Xu, et al. The study of optimal oxidation time and different temperatures for high quality VO_2 thin film based on the sputtering oxidation coupling method. Applied Surface Science **257** 8824–8827(2011)

3. Xiaofeng Xu, et al. The extremely narrow hysteresis width of phase transition in nanocrystalline VO_2 thin films with the flake grain structures. Applied Surface Science **261** 83–87(2012)

4. Jakub A. Koza, Zhen He, Andrew S. Miller, and Jay A. Switzer， Resistance Switching in Electrodeposited VO_2 Thin Films, Chem. Mater. **23** 4105–4108(2011)

5. Changhyun Ko, Zheng Yang, and Shriram Ramanathan. Work Function of Vanadium Dioxide Thin Films Across the Metal-Insulator Transition and the Role of Surface Nonstoichiometry, Appl. Mater. Interfaces **3** 3396–3401(2011)

6. Jiang Wei, Heng Ji, Wenhua Guo, Andriy H. Nevidomskyy & Douglas Natelson Hydrogen stabilization of metallic vanadium dioxide in single-crystal nanobeams. Nature Nanotechnology **7** 357–362 (2012)

7. John P. Perdew, Kieron Burke, and Matthias Ernzerh. Generalized Gradient Approximation Made Simple. Phys. Rev. Lett. **77** 3865–3868 (1996)

8. Xun Yuan, Yubo Zhang, Tesfaye A. Abtew, Peihong Zhang, and Wenqing Zhang, VO_2: Orbital competition, magnetism, and phase stability. Phys. Rev. B **86** 235103 (2012)

9. Wentzcovitch, Renata M.; Schulz, Werner W.; Allen, Philip B. VO_2: Peierls or Mott-Hubbard? A view from band theory. Phys. Rev. Lett. **72** 3389–3392 (1994)

10. Tao Yao, Xiaodong Zhang, Zhihu Sun, Shoujie Liu, Yuanyuan Huang, Yi Xie, Changzheng Wu, Xun Yuan, Wenqing Zhang, Ziyu Wu, Guoqiang Pan, Fengchun Hu, Lihui Wu, Qinghua Liu, Shiqiang Wei. Understanding the Nature of the Kinetic Process in a VO_2 Metal-Insulator Transition. Phys. Rev. Lett. **105** 226405 (2010)

11. J Cao, Y Gu, W Fan, L. Q. Chen, D. F. Ogletree, K Chen, N. Tamura, M. Kunz, C. Barrett, J. Seidel, J. Wu. Extended Mapping and Exploration of the Vanadium Dioxide Stress-Temperature Phase Diagram. Nano Lett. **10** 2667–2673 (2010)
